# ‘Most people have no idea what autism is’: Unpacking autism disclosure using social media analysis

**DOI:** 10.1177/13623613231192133

**Published:** 2023-08-22

**Authors:** Chris Edwards, Abigail M A Love, Sandra C Jones, Ru Ying Cai, Boyd Thai Hoang Nguyen, Vicki Gibbs

**Affiliations:** 1 Autism Spectrum Australia, Australia; 2 Griffith University, Australia; 3 Australian Catholic University, Australia; 4 Queensland University of Technology, Australia; 5 University of Sydney, Australia

**Keywords:** autistic adults, disclosure, discrimination, qualitative research, social media, stigma

## Abstract

Autism disclosure can be a complicated decision that autistic people experience. Positive outcomes can include feelings of acceptance and support, but negative outcomes can include stigma and discrimination. Although a surge in research on this topic has led to more understanding around autism disclosure, the methodologies used may have limited who was contributing to the conversation and data. To overcome this, we analyzed 3 years (2020−2022) of social media data (Reddit and Twitter) as this was public information that did not rely on researcher data collection. Reflexive thematic analysis of 3121 posts led to the generation of four themes: People do not understand autism (with experiences related to employment, dating, healthcare and mental health), autistic people just want privacy and respect, autistic people can lead us forward and non-autistic people need to assume more responsibility. We discuss how autistic adults experience the impact of society’s lack of understanding of autism on a daily basis whether they disclose or not, and that it is everybody’s responsibility to challenge negative stereotypes and promote a more inclusive society.

**Lay abstract:**

Autism disclosure – that is sharing their autism diagnosis or identity with a person or people – is a difficult decision for many autistic people. While telling people they are autistic can be positive and helpful, it can also create a lot of problems. What we have learnt is that disclosure is really complicated. Rather than asking research participants questions about what might happen, we looked at what people were saying on public social media posts (Reddit and Twitter) about what did happen. We used three years of posts that were related to autism disclosure from a wide range of adults (autistic and non-autistic). Four main ideas were created from our data, with the key finding being that society does not understand autism. This lack of understanding creates problems for autistic people in work, dating, healthcare and mental health. The remaining ideas were that autistic people should have privacy and be treated with respect, that autistic representation can help society and that non-autistic people need to do more to help autistic people. Our findings support that society needs to do more through autism advocacy, better media representation and more public role models. Increasing the accuracy of understanding of autism across society will mean that autistic people can feel safer to disclose if they want to.

Autism disclosure, or choosing to tell others that they are autistic, can be one of the most complex decisions autistic people experience (Botha et al., 2020). Disclosure can result in increased understanding, acceptance, support or strengthened relationships, but it may also expose the autistic person to being dismissed, judged or misunderstood (Huang et al., 2022; Lindsay et al., 2019; Love et al., 2023; Romualdez, Heasman, et al., 2021; Thompson-Hodgetts et al., 2020). Given the potential risks around disclosing, some autistic people are selective in their disclosure and many engage in ‘camouflaging’ or ‘masking’ to hide who they are (Cage & Troxell-Whitman, 2019; Cook et al., 2021; Huang et al., 2022). While concealing parts of their identity can reduce the possibility of stigma and discrimination, it can increase the risk of mental health difficulties (Cage & Troxell-Whitman, 2019; Cook et al., 2021; Han et al., 2021; Hull et al., 2017). Furthermore, non-disclosure may mean that an autistic person does not receive the support and adjustments that they need to thrive, particularly in higher education and employment (e.g. Cai & Richdale, 2016; Lindsay et al., 2019).

Outcomes following the disclosure of autism from the perspectives of both autistic and non-autistic people have been investigated across several contexts. A recent scoping review found that studies investigating disclosure based on hypothetical reactions of non-autistic participants presented with fictional scenarios were generally positive, whereas those from studies based on actual accounts of autistic people were more likely to be negative (Thompson-Hodgetts et al., 2020). Employment settings have been a particular focus of disclosure research because of the potential value of workplace adjustments and support for autistic adults (e.g. Lindsay et al., 2019; Romualdez, Heasman, et al., 2021; Romualdez, Walker, & Remington, 2021). In their systematic review, Lindsay and colleagues (2019) noted that disclosure can provide benefits beyond accommodations and support, extending to feelings of acceptance, inclusion and increased autism awareness. Workplace disclosure can also lead to improvements in an autistic person’s mental health and well-being (Romualdez, Heasman, et al., 2021). In contrast, negative disclosure experiences in the workplace can include stigma, bullying, discrimination, recruitment disadvantages and perceptions of inadequate support, understanding, acceptance and/or adjustments (Lindsay et al., 2019; Romualdez, Heasman, et al., 2021; Romualdez, Walker, & Remington, 2021).

These reviews highlight the complexity, risks and contradictions associated with disclosure, with calls for future research to consider real-life disclosure experiences across contexts (including workplace experiences beyond initial hiring) and with a more diverse population (Lindsay et al., 2019; Thompson-Hodgetts et al., 2020). Investigations into autism disclosure have traditionally used survey research, interviewing or focus groups. While there are distinctive benefits to each of these methodologies, each requires recruitment into research studies which can often lead to bias in who has access to, and who chooses to respond to, recruitment calls (Rubenstein & Furnier, 2020). In addition, questions in surveys and interviews are framed by researchers, again limiting the scope of the information that can be gathered (Lloyd et al., 2019). To address these limitations, Love and colleagues (2023) utilized a smartphone application to record real-time disclosure and non-disclosure experiences of autistic adults over a 2-month period. Their findings detailed the decision-making considerations and varying reactions that autistic adults experienced from others on a day-to-day basis. While there were strengths to this design, it also relied on recruiting participants who may have been drawn to disclosure/advocacy-type research and may only reflect a small piece of the wider autistic community (Lloyd et al., 2019).

One emerging method that allows researchers to capture data from a diverse sample without researcher involvement or reliance on recruitment is the analysis of publicly available social media data. Autism researchers have begun turning to social media data across a diverse range of topics including autism identification and diagnosis (autism blogs; Harmens et al., 2022), aloneness and connectedness (autism blogs; Petty et al., 2023), identity (Twitter; Egner, 2022), burnout (Wrong Planet and Twitter; Mantzalas et al., 2022), vaccine controversy (Reddit and Twitter; Jang et al., 2019) and therapy perspectives (Facebook; Sterman et al., 2022). This method can provide direct access to the rich lived experiences of autistic people and their communities, with data gathered across an extended period (Egner, 2022; Harmens et al., 2022; Mantzalas et al., 2022; Sterman et al., 2022). Furthermore, this naturalistic approach to collecting data minimizes researcher involvement and allows online conversations to happen naturally with people writing about what matters to them (Harmens et al., 2022; Sterman et al., 2022). It also provides the opportunity to hear from multiple stakeholders (e.g. autistic people, parents, carers and non-autistic individuals) on an international scale, particularly the inclusion of autistic users who may be more comfortable communicating online (Dekker, 2020; Egner, 2022; Gillespie-Lynch et al., 2014).

While there are strengths to social media data, there are limitations as with any methodology. For instance, the demographic characteristics of users can be unclear and/or difficult to obtain (Proferes et al., 2021). Furthermore, it has been argued that social media data may oversample more privileged people (e.g. technological skills, Internet skills and higher socioeconomic status) and ignore the views of the less privileged (Hargittai, 2020). Therefore, it is important that such potential biases from the data are acknowledged. Despite these limitations, researchers have demonstrated that social media data can be high-quality data that are of a similar standard to traditional research methods (Jamnik & Lane, 2017).

Users engage with platforms (e.g. Facebook, Twitter, Reddit and Wrong Planet) for varying reasons; however, Twitter and Reddit are the most popular platforms to discuss public issues which may be due to their open and accessible nature (Jang et al., 2019) with Reddit having a larger number of active users per month (Dixon, 2022a, 2022b) and Twitter emerging as a key platform for autistic voices (e.g. Egner, 2022). In order to complement previous autism disclosure research and contribute further to the understanding of the implications of autism disclosure for autistic people, we used posts from Twitter and Reddit to address the primary research question guiding this study: What do autistic adults and autism stakeholders express about autism disclosure in their social media posts on Twitter and Reddit?

## Methods

### Data sources

Public data were collected from Twitter (microblogging and social networking service, www.twitter.com) and Reddit (social news aggregation, content rating and discussion website, www.reddit.com). ‘Tweets’ (or Twitter posts) are limited to up to 280 characters where users can share posts with their followers and the wider public. In contrast, Reddit has no limit to the size of a post and includes a network of communities or ‘subreddits’ (e.g. r/autism) which allow like-minded individuals to connect over shared interests. For example, a Reddit post could include a topic such as ‘Ways to keep safe after disclosing autism diagnosis?’ followed by the user sharing as little or as much detail as they would like in the body section.

There are ethical considerations with researchers using public data on social media platforms while at the same time respecting confidentiality and consent, such as whether this use aligns with the users’ expectations when they registered (Proferes et al., 2021). Our choice of platforms was therefore deliberate as, unlike platforms such as Facebook which have user restrictions on who can see posts, users of Reddit and Twitter are made aware of the public and accessible nature of their posts when registering, regardless of whether the viewer has an account or not. The ethical components of this research were approved by the University of Sydney Human Research Ethics Committee.

### Reddit and Twitter users

Users shared a public post on Twitter or Reddit related to the disclosure of an autism diagnosis/autistic identity between the period 01/01/2020 and 01/10/2022. We selected this period as we wanted to capture the most recent posts around autism disclosure while still ensuring a comprehensive dataset. Given the public and accessible nature of their posts, we have chosen the term ‘user’ as their registration was with the platform rather than being a research ‘participant’. This included autistic people themselves and relevant stakeholders (e.g. a user sharing a disclosure experience of an autistic friend or a parent sharing an experience of their autistic adult (child) disclosing for themselves). While the posts were public, privacy regulations prevented us from collecting any demographic information from users. Users were assumed to be adults (18+ years) unless there was a clear indicator that they were younger (e.g. a reference to primary or secondary school), in which case their data were excluded.

### Procedure

**Figure 1.**
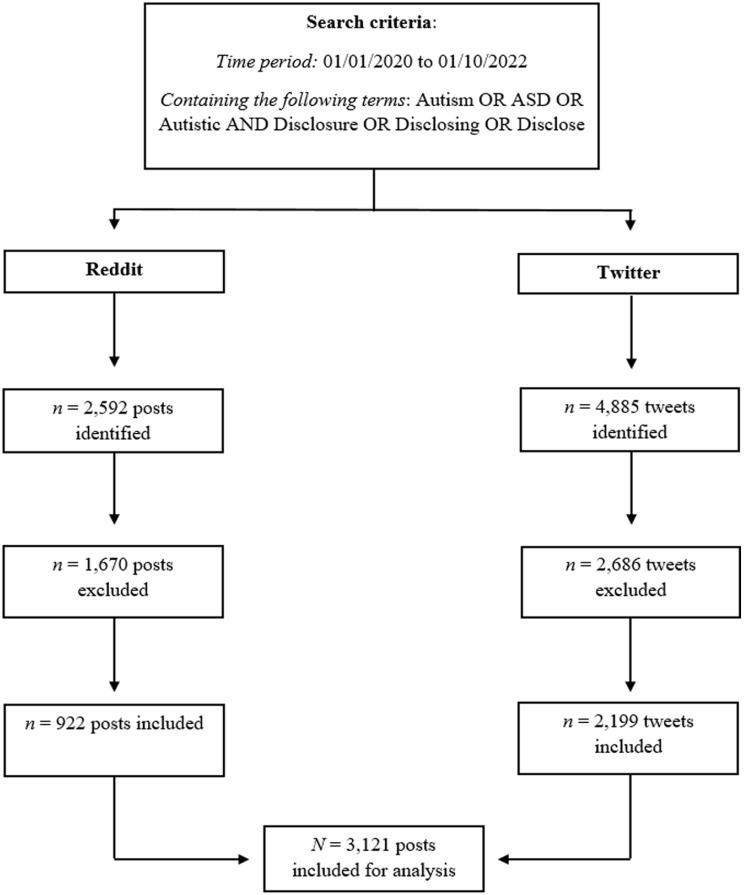
Data collection and cleaning process. *Note.* The Reddit dataset contains submissions matching the keywords (either in the title, self-text, subreddit or domain), created within the specified date range. The Twitter dataset includes tweets (including the ‘quote’ part of ‘Quote Tweets’) containing the keywords in their text, created within the specified date range. Retweets were excluded from this dataset.

The first author (C.E.) engaged with the Australian Digital Observatory (B.T.H.N.) to provide expertise around the data collection and analysis process. They conducted a feasibility analysis using data from the Australian Twittersphere (Bruns et al., 2014) and Reddit to assess the volume and relevance of data based on the initial search criteria (‘Autism OR ASD OR Autistic AND Disclosure OR Disclose or Disclosing’). Following the feasibility analysis, data were collected globally from Twitter using Twitter API v2 (Academic Research Track), and through a combination of Reddit’s official API and Pushshift API (Baumgartner et al., 2020), and provided to the team in a CSV format (Microsoft Excel). A summary of the data that were identified, excluded and included for analysis is shown in Figure 1.

### Data cleaning

Two researchers (C.E. and A.M.A.L.) met repeatedly to discuss the data cleaning for both sources to ensure a consistent approach to what was included and excluded. A draft list of exclusion criteria was developed based on ethical considerations and their understanding of disclosure. Duplicate posts were removed using Excel’s Duplicate Values function. Symbols and extra spaces were also deleted to enhance readability. C.E. cleaned the first 100 tweets, identifying tweets to be included, excluded and flagging items where he was unsure. A.M.A.L. reviewed these tweets in order to gain agreement on the included/excluded tweets. Any discrepancies were resolved through discussion. A similar process was used for the Reddit posts. This process was repeated once more before the researchers commenced cleaning the remainder of the data. The finalized exclusion criteria agreed upon by both researchers were as follows:

- Content was not relevant to autism disclosure (e.g. an autistic person’s disclosure related to mental health).
- Content could not be understood (e.g. very limited narrative, mostly hashtags, not written in English or it required clicking an external link for context).
- Content indicated that the user was under the age of 18 years (e.g. a primary or secondary school reference).
- Content was related to autism disclosure research.
- Content was marketing/self-promotional (e.g. an organization promoting a disclosure resource).
- Content related to parental disclosure (e.g. a parent disclosing on behalf of their minor child).

### Data analysis

The qualitative data analysis was guided by Braun and Clarke’s (2006, 2019) reflexive thematic analysis, using an inductive approach to produce codes that were reflective of the data. As this qualitative approach emphasizes the active role of the researcher in knowledge production and views this as a resource (Braun & Clarke, 2013), this was completed by an autistic researcher (C.E.) to better understand the meaning behind the autism disclosure experiences shared by users and both semantic and latent coding were utilized.

Data were imported into NVivo for initial coding while considering the overarching research question. As the research question was specifically focused on social media posts that referred to autism disclosure, these were analyzed independently of the surrounding conversation. By focusing on individual posts rather than the broader context, this allowed us to explore themes and meanings without being influenced by the dynamics and biases of the overall conversation. Codes were continuously revised by the first author through multiple iterations of coding and identifying patterns. A set of potential themes and subthemes were defined and named, and compared with the coded data items and the entire dataset. During this process, the research team met repeatedly to review the candidate themes and subthemes to ensure that they accurately reflected the data and answered the research question.

### Community involvement statement

This research was conducted by the Aspect Research Centre for Autism Practice, a participatory research centre that includes autistic and non-autistic researchers. In addition, the research team partnered with an autistic autism researcher (S.C.J.) with over 20 years’ experience in academia in addition to being an autism advocate and parent of two autistic adults. The principal investigator of this study (C.E.) is an early career autistic autism researcher with a background in psychology and education. Other team members included autism researchers who were also a parent (V.G.) and sibling (A.M.A.L.) of an autistic adult and an autism researcher (R.Y.C.). This study reflected a co-produced model of research practice, with a diverse team of autistic and non-autistic researchers with experience across education, psychology and health seeking to answer the same question (den Houting, 2021).

## Results

**Figure 2.**
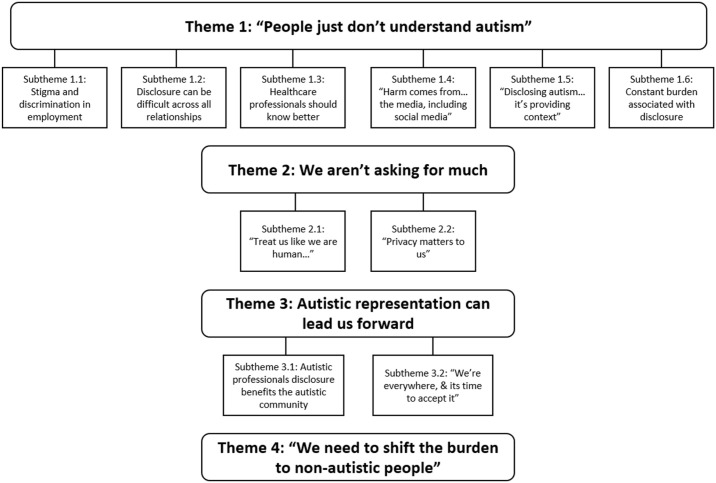
Thematic map of themes and subthemes from Reddit and Twitter. *Note.* Theme 1 was both a stand-alone theme and the overarching theme in our findings.

Figure 2 presents a thematic map detailing the themes and subthemes. The quotes in this section have been reproduced verbatim, including their corresponding data source and entry number of the dataset (e.g. R414 is data entry 414/922 of the Reddit data), and may contain profanities and grammatical errors. Supplementary Material containing quotes supporting the themes and subthemes has also been included.

### Theme 1: ‘people just don’t understand autism’

Analysis of both Reddit and Twitter posts indicated that ‘ASD is poorly understood’ (T1986) across society. ‘Most people have no idea what autism is’ (T430) with autistic users sharing that their identity was commonly questioned with people ‘thinking that I’m lying or faking (autism) for attention’ (R275). Users shared that because of the way autism is viewed in society, autistic people ‘HAVE to consider potential implications of telling people’ (R323). Autistic users expressed that they were hesitant to disclose, with one user noting that ‘the few times I have told others I’ve been bombarded with “cures”. Use this oil, do this detox, talk to this natural doctor, buy these crystals, it’s always overwhelming’ (R741). The widespread stigma associated with autism in society was described by one user as ‘HUGE’ and in their own experiences, they had been called, ‘Rain Man, a curse, a savage’ and even ‘compared to CANCER’ (T1126) after their disclosure.

#### Subtheme 1.1: stigma and discrimination in employment

Workplace discrimination following disclosure was the most frequent topic shared by users, where autism disclosure was described as a barrier to employment, a cause of workplace bullying and a reason behind the loss of a job. Many users shared that their disclosure during a job interview led to an immediate rejection, ‘another job down the gurgler (drain)’ (R620). They wrote about how they could visually see their potential employer’s ‘estimation of me drop like a stone’ (T2172). Many viewed their success in the job market ‘as how well we can pretend we’re like non-autistic people . . . or whether our non-autistic employers see us as human’ (T1728). As a result, many chose not to disclose as they could not ‘get anywhere disclosing my autism. I only ever got into places by hiding it’ (T1643).

For autistic people in employment, there was reluctance to disclose due to stigmatizing views expressed by co-workers such as ‘eww, I would never want to work with someone like that’ (T1553) and subsequent discrimination from colleagues and employers. Some co-workers were described as bullying autistic staff by ‘being cruel and harassing him in some way’ (R836), and others questioned their identity, ‘yeah, but you’re not REALLY autistic, are you?’ (T2038). In other instances, autistic users detailed that they lost responsibilities in their roles after disclosing despite having the necessary certifications, such as being told they were ‘a risk to the safety of customers and associates’ (R618).

Users wrote about how discrimination and bullying following disclosure often led to them being ‘bullied out of a job’ (T690) or otherwise being ‘fired . . . for disclosing’ (T703). Users indicated that employers lacked an understanding of autism. Human resources staff would often state ‘something so completely ignorant and false’ (R283), even thinking that ‘autistic people are “brain damaged”’ (T2157). Some users revealed they lost their job immediately after disclosing; ‘fired from my job with no warning. . .’ (R719). Others lasted a few days, ‘it took them three days from when I submitted my autism paper, to coming to the conclusion to dismiss me’ (R211) and some a few months, ‘was abused, ostracized and within the year had been relieved of my job I’d been flying in for four years’ (T1322).

#### Subtheme 1.2: disclosure can be difficult across all relationships

This subtheme describes how hard disclosure to friends, family and potential/current intimate partners can be. Many users indicated that disclosing harmed their close friendships such as being ‘ripped to shreds by a friend of 10 years after disclosing my autism’ (T2047). In other instances, disclosure ended friendships, ‘all of my “friends” blocked me and kicked me out of their lives because they found out I was autistic . . . and one of them told me “we don’t want to be with someone retarded”’ (R513). Users commonly expressed that it ‘depends on one’s family. It may not be safe for some autistic people to disclose. In other situations, it is totally fine . . . I know instances where it has ended family relationships’ (T91).

The complexities and challenges of disclosing with intimate partners were the most common relationship topic shared by users. Although many expressed an interest in intimate relationships, some had ‘kinda given up on love, whenever I disclose I have autism to a girl I’m dating, I get ghosted’ (R753). Similar to the experiences in employment, prospective partners were described as uneducated about autism, ‘autism isn’t contagious. Yes, I have had a date ask me that once upon me disclosing my autism’ (T1522). Disclosing frequently led to discrimination and the immediate end of relationships, such as one user who received ‘I can’t handle that’ (T1941) and was immediately left during the date. This was even the case in established relationships, with some users indicating they would never ‘tell my significant other that I have autism, not even on my death bed’ (R722).

#### Subtheme 1.3: healthcare professionals should know better

Disclosing to a healthcare professional can ‘contextualize my experiences, should inform medication decisions, as well as other health implications’ (T1069), but users shared they often ‘worry it will result in a lower standard of care’ (T829). Negative experiences were common and occurred across a range of health professionals (e.g. general practitioners, psychiatrists, nurses, paramedics, audiologists, pharmacists and counsellors) who often demonstrated a lack of understanding of autism. This included not being believed by general practitioners, ‘you (autistic)? Impossible’ (T1525) and ‘you’re not autistic . . . we’ve been talking without an issue’ (R683). Posts referring to negative experiences with nurses were also common such, ‘every time I disclose I’m autistic to a nurse they always argue with me??? Like they take it as an invitation to debate my diagnosis’ (T87). There were instances of infantilization, ‘being treated like a fucking moron while in a full-blown autistic meltdown by these effing paramedics’ (R699). Users also emphasized a lack of autism knowledge in critical care contexts such as an emergency department, where an autistic friend of one user was told during intake ‘you can’t have autism, if you were autistic you wouldn’t be able to tell me’ (R739).

#### Subtheme 1.4: ‘harm comes from . . . the media, including social media’

The way that the media portrays and discusses autism contributed to a reluctance to disclose. Several users wrote about how impactful it was when the media portrayed school shooters who were autistic, ‘the media likes to trot out the diagnosis in relation to school shooters . . . disclosing your diagnosis could put you in more danger, not less’ (T2011). Another user noted how their social media and the news were flooded with comments such as ‘all people with autism are MONSTERS!!!’ following a shooting incident (R546). The media’s portrayal of autism led to concerns for safety due to misinformation and inaccurate or incomplete portrayals of autistic people and this contributed to their ‘motive for non-disclosure’ (R546).

In addition, the way in which autistic people were portrayed in film and television impacted disclosure decisions as one user noted how these (in reference to Sia’s 2021 film, Music) portrayals made them ‘feel infantilized and stereotyped . . . This is why I STILL have issues embracing my autism and disclosing to others’ (T1630). The long-lasting impact of inaccurate portrayals was evidenced by numerous Rain Man references despite the film being released in 1988, ‘I can’t count the number of insensitive, ill-informed reactions to me disclosing I’m autistic as a result of bad depictions’ (T1744). This includes television references where there is concern that following disclosure people ‘will immediately think oh, like *Love on the Spectrum* or *Atypical*’ (R63). Other common television references included *The Big Bang Theory*, such as one autistic user who was bullied in the workplace with comments such as ‘it’s like dealing with Sheldon Cooper’ (R82).

#### Subtheme 1.5: ‘disclosing autism . . . it’s providing context’

On a positive note, disclosure was viewed as a way of helping people ‘understand about me . . . it would be very beneficial if they understand my brain works a little differently’ (R24). This included explaining their behaviour, so ‘people are less likely to think I’m being intentionally rude or lazy – which matters a lot’ (T113). This was common in the workplace, as telling others that they are autistic ‘would maximize my ability to work effectively with others in the lab and would best “help me help them” as a result’ (R28). Users also considered disclosure as a way of ‘providing context to our communications so we may work on understanding each other better’ (T29).

#### Subtheme 1.6: constant burden associated with disclosure

Autistic users wrote about the emotional impact, particularly the fear and anxiety associated with the idea of disclosure:

I don’t want to be the autistic recluse, but I’m honestly scared of the world. I don’t want to disclose my diagnosis to people whom I need to in order to receive accommodation because I’m scared of what will happen. I’m scared of being judged yet again. I’m scared of being discriminated against. I’m scared that someone will call me a poorly raised child, even though my parents raised me the right way. I’m scared I’m going to be another face on the nightly news (R793).

This emotional burden was prominent among users in higher education as well as employment settings, where there was fear that disclosure would lead to ‘fewer job opportunities’ (R627) or that it would put the ‘brakes on career advancement’ (R616). Other users expressed how they needed support and accommodations to thrive at work but felt that it was ‘impossible to request accommodations for autism without being treated like a child or murderer’ (T1476). This included parallels between disclosure and ‘professional suicide’ (T770 and T1805) or ‘employment suicide’ (T795). As a method of trying to manage this burden, ‘I mask hard . . . the effort to mask destroys me. But at least I’m seen as an equal intellectually’ (T1441). But masking can take a toll, and when one user ‘couldn’t mask any more . . . I disclosed and was bullied out of the job and it put me in hospital’ (T1577).

### Theme 2: we are not asking for much

Given the negative views and experiences described in Theme 1, it is perhaps unsurprising that users consistently urged others to treat them with respect. They expressed a strong desire for their privacy to be respected and valued, as well as for respectful and dignified treatment.

#### Subtheme 2.1: ‘treat us like we are human’

Regardless of whether autistic users disclose or not, they want others to ‘treat us like we are human’ (T824). Some users indicated they were bullied when they ‘don’t disclose’, often because they were seen as ‘the most vulnerable person in the office and the easiest target’ (R693). In response to bullying, there was a pressure to disclose, but ‘I shouldn’t have to disclose that I’m autistic for you to treat me with basic respect and dignity’ (T1156). Users shared that they did not like feeling forced or pressured to disclose to ‘gain the privilege of being treated like a human being’ (R674), even comparing disclosure with holding ‘a white flag to prevent bullying’ (T1687).

However, disclosing often made the situation worse. Autistic users commonly wrote about how disclosure led to experiences such as being ‘dehumanized and treated like you’re disposable trash’ (R671). Other users described the frustration of infantilization, how people ‘suddenly change and start treating me as if I’m stupid or talking to me like a child’ (R103). In one instance, a user shared how their autistic colleague was ‘frequently bullied. People called him creepy, annoying, and awkward . . . then he disclosed and they all started handling him with kid gloves (while still talking behind his back obviously)’ (R738). While many autistic users specifically avoided disclosure as they did not ‘want to be talked to like a child’ (R85), many posts indicated that ‘either way I lose’ (T699).

#### Subtheme 2.2: ‘privacy matters to us’

Autistic users commonly stated that disclosure was a personal and private choice, not to be forced, not to be non-consensual, ‘just cause YOU think it’s fine doesn’t mean it is’ (T1240). For instance, in response to a grandparent oversharing with new neighbours, an autistic user was left ‘completely distraught . . . she didn’t have any right disclosing . . . It’s absolutely humiliating and I hate this so much’ (R531). These experiences were common in workplaces. In some cases, co-workers shared this information ‘to my manager without my knowledge’ (R356). In other instances, it was the manager in the wrong despite their good intentions, ‘my boss, in an effort to help, disclosed the fact that I am autistic, to the ENTIRE BUILDING STAFF’ (R453). This decision took away agency and privacy from the autistic user, ‘my new employer just told the team I had autism . . . but fuck. Pretty sure disclosing my disability isn’t legal. I wouldn’t have chosen to disclose to the rest of the team’ (R371).

### Theme 3: autistic representation can lead us forward

On a positive note, some autistic professionals disclosed to their autistic patients/students/participants and reported that it was often received positively, with their lived experience being an ‘invaluable resource’ (R154). Users also spoke of disclosing for ground-level advocacy, as well as how autistic public figures can be the key to challenging negative stereotypes and as a solution to the challenges posed by misunderstandings and misconceptions of autism as described in Theme 1.

#### Subtheme 3.1: autistic professionals’ disclosure benefits the autistic community

Users expressed being drawn to roles that directly supported the autistic community and that their disclosure often added value to their role, ‘because nobody is going to understand an autistic person like another person on the spectrum’ (R100). Users wrote about how they would be ‘happy to have an autistic therapist’ (R12), but the ‘stigma is so bad that they don’t feel they can disclose’ (T1846). An autistic therapist shared that they disclosed when it ‘feels therapeutically beneficial’ (T383), and an autistic patient being aware of an autistic doctor is ‘what made me choose her’ (T1976). Users described the positive responses from disclosing in their roles supporting autistic people, where it is ‘great it is to have someone with so much insight’ (T1951) and that it explains why they ‘were good at your work with autistic kids’ (T945). Autistic educators also disclosed as they felt their experience meant ‘I can support her in the best way and I completely understand how to use stuff that works with our thinking’ (T756) and it can give ‘other autistic students hope that they can be successful’ (T39).

#### Subtheme 3.2: ‘we’re everywhere, & it’s time to accept it’

Users shared the value of disclosing as a form of autism advocacy, with autistic representation through public figures being a way to challenge stereotypes. Some users expressed that they ‘like to be an advocate and leader’ (R359) as it can ‘impact other people and also bring awareness’ (R52). While autistic users acknowledged the challenge and discrimination they would likely face, many saw disclosure as part of their ‘moral code’ (R359), and that ‘logically it’s the only way . . . it’s our duty’ (R498).

Beyond the ground-level advocacy, users shared that they were ‘grateful for each and every celebrity and public figure who chooses to disclose their autism. It’s good and necessary’ (T478). For example, while Elon Musk received mixed responses to his autism disclosure in our data, the positive comments from users included being ‘proud of him for bringing neurodiversity to the national stage!’ (T1401) and that it ‘gives hope to guys like me, and is very much appreciated’ (T1426). Further praise for the disclosure of public figures included celebrities such as Anthony Hopkins, Wentworth Miller, Melanie Sykes and Julia Fox and that ‘we love to see it’ (T6). Users also expressed a mutual desire for more autistic role models, ‘autistic youth need autistic adult mentors, teachers, coaches, guidance’ (T134) and that ‘those of us who can disclose make it easier for those who come after us’ (T1719). Users also shared a view that ‘I hope more successful autistic people do come out in the future to help break this harmful stereotype’ (R484).

### Theme 4: ‘we need to shift the burden to non-autistic people’

While the previous themes represented a shared narrative between Reddit and Twitter, Theme 4 reflected a theme primarily derived from Twitter users; ‘we need to shift the burden to non-autistic people’ (T1144) when hoping for change around disclosure and that ‘organizations, rather than autistic individuals need to take more responsibility for facilitating disclosure and improve outcomes to it’ (T1305). Workplaces in particular can be a safer place to disclose if they were ‘less of a sensory hell-zone. That works for everyone, not just us’ (T481). In fact, ‘if workplace systems and processes were designed or adapted to be neurodivergent inclusive then personal disclosure would be less necessary’ (T721). The burden of disclosure can be shifted by normalizing ‘all of these accommodations . . . (then) there is no need for a person to disclose’ (T282). This existed in some workplaces. For instance, one user did not disclose at a new job as they were ‘worried about discrimination, and then because I . . . didn’t need to. Every accommodation that I asked for was simply a request and was granted’ (T852). In another case, ‘my employer has accommodated my ADHD and autism without me ever even disclosing either . . . They just do what is needed to support their employees’ (T830).

## Discussion

This study aimed to extend what we know about autism disclosure by analyzing recent (2020−2022) public posts from Reddit and Twitter. Social media is an established platform for individuals to share stories and perspectives about autism and is an emerging data source for researchers who aim to seek data from a representational and comprehensive source (Bakombo et al., 2023). The primary finding from our thematic analysis was that *People just don’t understand autism* and this lack of understanding formed the basis for the remaining themes.

When telling others about being autistic or considering disclosure, users consistently described a lack of understanding of autism (*Theme 1)* that extended to stigmatizing and discriminatory responses from others. These were shared during encounters across all aspects of their lives including employment, healthcare, dating, friends and family relationships, even contributing to additional mental health concerns. Our findings provide further evidence of the stigmatized social status of autistic people (Botha & Frost, 2020) and are consistent with previous research where autistic people have reported experiencing stigma (Botha et al., 2020; Shtayermman, 2009) and are perceived negatively, even in brief interactions, by non-autistic people (Sasson et al., 2017). Users on both Reddit and Twitter often turned to social media for advice regarding their hesitation to disclose or their negative experience around disclosure due to the impact of inaccurate or negative portrayals of autism. It is known that depictions of autism in the public sphere may promote negative stereotypes and stigmatization (e.g. Holton et al., 2014; Jones et al., 2023), and our study demonstrated that these inaccuracies influence disclosure decisions for autistic individuals. Therefore, to improve experiences of disclosure for autistic individuals, accurate knowledge of autism and acceptance of autism among community members is a critical step in combating the systemic barriers and prejudice faced by autistic individuals in various domains of their lives.

We also found that a lack of understanding about autism leads to fear and anxiety around anticipated autism disclosure or, for others, negative consequences following an actual disclosure experience. This lack of understanding about autism is extremely problematic within employment contexts, a consistent finding in other autism disclosure work (Huang et al., 2022; Johnson & Joshi, 2016; Lindsay et al., 2019; Love et al., 2023; Romualdez, Heasman, et al., 2021; Romualdez, Walker, & Remington, 2021). We were able to capture workplace disclosure outcomes beyond initial experiences by detailing the discrimination that autistic people encountered over time in employment settings, particularly highlighting how common it was for autistic users to share that they felt disclosure led to the loss of job. This is an important consideration as previous research has implied autistic people should disclose in order to access accommodations and support (e.g. Thompson-Hodgetts et al., 2020), yet our findings suggest that this can this may come with risks for the autistic person. While the hiring process has been well documented as a barrier to autistic workers (Davies et al., 2023; Finn et al., 2023), our findings emphasize the urgent requirement for organizations to become more inclusive at every staff level to support autistic employees across their employment journey.

In addition to employment contexts, autistic and non-autistic users in our sample detailed numerous poor disclosure experiences with varying healthcare professionals. This is perhaps unsurprising as health professionals often report a lack of knowledge and confidence in supporting autistic people (e.g. Corden et al., 2021). However, this is an area that requires immediate attention to ensure equity of healthcare access for autistic individuals. Given the increased prevalence of co-occurring conditions in the autistic community (Lai et al., 2019), this is particularly important as disclosure can be key to quality healthcare. Notably, our findings also found that autistic professionals (e.g. in healthcare and education) often felt safe disclosing to their autistic patients/students and these disclosures were often received positively with the recipient reported to value the person’s lived experience in the role. Some users even sought out openly autistic healthcare professionals, while others wished there were more.

These findings are consistent with the perspectives of psychologists regarding the valuable contributions that autistic psychologists and therapists can offer in their support and understanding of their autistic clients (Jellett & Flower, 2023; Muggleton et al., 2022). However, these findings also underscore the significance of mitigating the stigma associated with autism within these compassionate professions. By doing so, it becomes possible to enhance safety and create opportunities for disclosure, as these circumstances represent instances where autistic individuals are particularly vulnerable. Moreover, other fields have also acknowledged the advantages of involving autistic professionals in providing support to the autistic community, including the implementation of peer support initiatives (e.g. Crane et al., 2021; Gillespie-Lynch et al., 2021; Taylor, 2021). Consequently, these implications hold particular relevance for organizations directly involved in assisting the autistic community, as the promotion of inclusive environments wherein autistic staff members feel safe enough to disclose their identities can yield significant benefits for both staff and the autistic individuals they serve as participants, patients or students.

Importantly, our findings draw attention to the emotional burden felt by autistic individuals as they consider autism disclosure opportunities, and oftentimes, to the negative consequences that result from disclosure experiences. While previous research has highlighted the association between disclosure and camouflaging or masking (Cage & Troxell-Whitman, 2020; Cook et al., 2021), our findings extended this by capturing the costs of camouflaging to avoid disclosure and the constant fear and anxiety associated with disclosure decisions and outcomes. For users in our study, the lack of understanding of autism was directly related to this emotional burden and is a likely contributor to the increased rate of mental health concerns among autistic people compared with non-autistic people (Lai et al., 2019). This overwhelming impact highlights how critical it is to promote a more inclusive society where autistic people do not feel the need to live with constant fear and anxiety around autism disclosure.

Users in our study suggested positive media portrayals and more autism advocacy through public figures could be two ways to ensure a safer environment for autistic people who want to disclose or be more authentic in their communities. This aligns with emerging research that shows mass media is often considered the most prominent source of information on conditions such as autism for the general public, but the usual lens through which it is portrayed (skewed and often negative) is problematic and promotes stigma (Fontes & Pino-Juste, 2022; Jones et al., 2023). Therefore, the media must consider more diversity in autistic portrayals, more ethical and accurate reporting, and include relevant strengths rather than focusing on weaknesses. For instance, television and movies need to include more realistic autistic characters to educate society about what autism is, such as Quinn Gallagher-Jones (*Heartbreak High*) rather than Sheldon Cooper (*The Big Bang Theory*). Furthermore, the more autistic people who feel they are in positions to be open about their identity, their ability to challenge stigma and educate society about autism can create a safer world for others who may want to disclose in the future.

### Strengths and limitations

This research was strengthened by the amount of rich autism disclosure information that was publicly available on Reddit and Twitter. This method provided us the ability to collect thousands of posts on an international scale from a diverse range of autism stakeholders. The findings of our research show that, without prompts from surveys or interviews, the posts around autism disclosure are focused on a need for more education, more respect, more awareness and more support from non-autistic people. These findings suggest that our users were different from previous study participants and research that may have attracted participants drawn to advocacy and, more importantly, those who felt their personal disclosure opportunities were more positive than negative (e.g. Farsinejad et al., 2022; Huang et al., 2022; Love et al., 2023). In addition, the research was strengthened by the co-production approach that was embedded from conception to dissemination.

While rich lived experience data were available, the retrospective nature of the data created limitations as there was no option to follow up with any users. Given the privacy regulations of Reddit and Twitter, we have no available demographic data to describe who the users were, other than they were able to write a post in English. It was often not possible to know whether the user was autistic or not unless self-reported in their post. However, this did not limit the quality of our research as wanted to examine any post related to autism disclosure in our time period. While many autistic people prefer online communication, it is possible this method of data collection excluded autistic adults who experience challenges with online media. In addition, while ‘disclosure’ is a term commonly used in autism research, not including lay search terms such as ‘sharing’ or ‘telling’ may have influenced who our users were. Research also suggests that social media users are more likely to write a post that reflects a negative experience rather than a positive one, which may have influenced our findings towards the negative disclosure experiences (Shahbaznezhad et al., 2020). Based on the above limitations, many of which are related to the analysis of social media data, the disclosure experiences discussed here should be interpreted with caution given it is difficult to know who the users were and who the experiences are more likely to apply to.

## Conclusion

This study is the first to examine autism disclosure using social media data and demonstrates how social media can be utilized to better understand the experiences and perspectives of autistic individuals. The impact of misinformation around autism is a direct call for society to improve social perceptions of autism. Research and support must target the social and structural factors that lead to stigma and discrimination. Autistic adults feel the impact of society’s lack of understanding of autism on a daily basis whether they disclose or not, with widespread stigma and experiences of discrimination across contexts such as employment, healthcare and relationships, with these experiences reducing their quality of life. Given the risks and private nature of disclosure, people must respect the confidentiality of an autistic person’s disclosure. The findings of this study are relevant for every member of society (e.g. researchers, media professionals and employers) as it is everybody’s responsibility to shift the burden from autistic people. It is everybody’s responsibility to reduce the spreading of misinformation about autism. It is everybody’s responsibility to create an inclusive society where autistic people can feel safe to disclose if they choose to.

## Supplemental Material

sj-docx-1-aut-10.1177_13623613231192133 – Supplemental material for ‘Most people have no idea what autism is’: Unpacking autism disclosure using social media analysis Supplemental material, sj-docx-1-aut-10.1177_13623613231192133 for ‘Most people have no idea what autism is’: Unpacking autism disclosure using social media analysis by Chris Edwards, Abigail M A Love, Sandra C Jones, Ru Ying Cai, Boyd Thai Hoang Nguyen and Vicki Gibbs in Autism
