# Supplementary material for: ‘Most people have no idea what autism is’: Unpacking autism disclosure using social media analysis
Source: Autism. 2023 Aug 22;28(5):1107–19. doi: 10.1177/13623613231192133 (PMC11067419; doi:10.1177/13623613231192133)
Supplement: sj-docx-1-aut-10.1177_13623613231192133 – Supplemental material for ‘Most people have no idea what autism is’: Unpacking autism disclosure using social media analysis [file sj-docx-1-aut-10.1177_13623613231192133.docx]

**Supplementary quotes to further illustrate themes and subthemes**

| **Theme 1: “People just don’t understand autism”** |
| --- |
| Subtheme 1.1: Stigma and discrimination in employment   1. EW! Those PEOPLE should not be allowed to work with the public. (R488) 2. Refused to allow me to enter the position I was hired for. (R713) 3. If I disclose it in an interview, I am immediately told I am not a good fit. If I disclose after, I am fired within the week. (T81) 4. This question comes up a lot, mainly because most #ActuallyAutistic staff get bullied, or fired, or don't fit in and get fired, or get fired for disclosing, or aren't supported, or can't get hired.... (T703)   Subtheme 1.2: Disclosure can be difficult across all relationships   1. Laughed in my face… she basically said that I’m being ableist to people who *actually* struggle with autism. (R83) 2. I’m 21F relatively attractive, very nice and sweet. No one will date me. The second I mention I have autism- they will not date me. When I don't mention autism on my profiles I get plenty of matches. The second I put autism in my bio, the matches stop. (R276) 3. He's hurt and angered that I didn't tell him I have ASD before starting our relationship, and he wants an apology. (R638) 4. I was talking to this girl on Tinder and she told me she worked with mentally disabled people so I told her I had autism and she was just like "okay" and unmatched from me. (R690)   Subtheme 1.3: Healthcare professionals should know better   1. Then there's the emergency services visits where us disclosing we're autistic get us either some "you don't look autistic, is it a real diagnosis? Who established it, when and where?" or they just stop listening and looking at us and start talking exclusively to our partner. (T1939) 2. "People with Autism don't feel emotions"… Those words branded into my skull forever, my first time trying to disclose to my family doctor. (T1828)   Subtheme 1.4: “Harm comes from… the media, including social media”   1. There is no positive stereotype of us portraying a guy who's struggling with social cues that isn't some fucking genius or whatever put in the media. (R671) 2. I've often hesitated to disclose my diagnosis on the spectrum. I fear invoking peoples' preconceived notions of autistic people, how we're all somehow Sheldon from The Big Bang Theory. These days I grow weary of the ongoing war on neurotypical people. (T248)   Subtheme 1.5: “Disclosing autism… it’s providing context”   1. I'm well aware that I do not have to disclose that I have autism, however - I want to. I'm sick of masking and I'm sick of people not understanding why I have to ask extra questions to understand context. (R385) 2. For example, if I disclose to people I am autistic, I do so with the aim of asserting my difference, hoping to have that difference affirmed, recognized, and accepted/accommodated. (T438) 3. I rather disclose my #autism than force eye contact. In my world, eye contact is for very close relationships only. There are other, less stressful ways to express that I'm a decent human being who has some manners. (T996)   Subtheme 1.6: Constant burden associated with disclosure   1. Don't feel safe to disclose that I'm autistic. It costs me to mask; but it would cost my career to disclose. (T1451) 2. Afraid they'll think I won't be a ‘fit’ parent. (R18) 3. I learned to survive by masking, and I owe modelling (mimicking others through observation) as a huge reason for the way I function today. Also motivated by the fear of patronization. My biggest fear has always been people treating me different because of my diagnosis. (R298) 4. I have nothing to show for my autism other than countless hours of studying and coming to my own realization. It's hard enough to be believed but also to get the accommodations I need to be successful. (R544) |
| **Theme 2: We aren’t asking for much**  Subtheme 2.1: “Treat us like we are human…”’   1. I'm terrified they would either invalidate me and tell me I don't fit their image of autism, or that they would start treating me differently. I want to be treated like anyone else, even if I know that I'm different. (R434) 2. I regret ever telling people I am autistic… And I think in some ways management thinks I need special treatment because of it, when really I just need to be treated like a human being. (R853)   Subtheme 2.2: “Privacy matters to us”   1. Went behind my back and told the managers about my autism after I helped a customer who was also autistic avoid a meltdown… told them I couldn't do my job. (R442) 2. I'm just so sick of the first thing they say about me being that I am autistic. It's not like I'm even a human to them. (R61) 3. That it’s my choice to disclose. (R703) 4. Diagnosis disclosure without consent, don’t do it. (T154)   **Theme 3: Autistic representation can lead us forward**  Subtheme 3.1: Autistic professionals disclosure benefits the autistic community   1. It’s a good thing for autistic participants to see someone autistic ‘thriving in a job that they like in an environment comfortable to them.’ (T1958) 2. I make it a point to disclose to my clients for this reason. It might not be the exact same experience but I have found it provides a better sense of trust and empathy. (T1680) 3. When I know a student is autistic and I see them struggling, then I tell them "you know, I'm autistic, and this is something that gives me trouble too." (T755)   Subtheme 3.2: “We’re everywhere – it’s time to accept it”   1. The nuanced manner in which '@wentworthmiller opened up about being #ActuallyAutistic sets such a good example for other (high profile) #Neurodivergent people who are still in doubt about how best to disclose. (T1152) 2. Refreshing to see an NHS clinician being publicly out as a disabled person. NHS staff are often hesitant about disclosing their disability status yet here's Ollie getting an autism diagnosis at 40 years old and seeing the advantages of that at work. (T93) 3. When prominent figures disclose their autism condition, the number of posts about autism increase… opportunity for trustworthy info about the condition and greater outreach. (T671) |
| **Theme 4: “We need to shift the burden to non-autistic people”**   1. I think often it is argued as an employee’s responsibility to come forward and disclose they need support. I think employers genuinely need to educate themselves more on Autism and ADHD and go the extra mile as inclusivity and accessibility go hand in hand. (T352) 2. It's wonderful when job interviews are enabling, instead of creating barriers. (T135) 3. We don't fault them, there is a lack of education. If you haven't heard from your #neurodivergent employees, your #culture is likely an unsafe place to disclose! (T238) 4. To say, "Of course we'll accommodate you IF YOU DISCLOSE," shows you have no clue what you're doing. (T549) 5. I don't like how typically the onus is on autistic people to disclose to initiate this. Employers should be promoting neurodiversity/acceptance. (T1999) |
